# Epignathus with oropharynx destruction

**DOI:** 10.4322/acr.2021.293

**Published:** 2021-06-25

**Authors:** Valerio Pellegrini, Francesco Colasurdo, Massimiliano Guerriero

**Affiliations:** 1 Ospedale Antonio Cardarelli, Regional Health Authority of Molise, Medical Department, Campobasso, Italy; 2 Ospedale Antonio Cardarelli, Regional Health Authority of Molise, Analysis Department of Transmural Services, Campobasso, Italy; 3 Ospedale Antonio Cardarelli, Regional Health Authority of Molise, Pathology Laboratory Department of Transmural Services, Campobasso, Italy

**Keywords:** Fetus, Pathology, Oral Teratoma

## Abstract

Epignathus, is a rare oropharyngeal teratoma arising from the head and neck region. Sporadic cases have been described with associated intracerebral teratoma. Even more infrequent and extraordinary is the circumstance of a teratoma with oropharynx destruction. We describe the case of a fetus with pharyngeal mass that completely destroyed the oral cavity. The histological examination revealed an immature teratoma (G3); only one other G3 case has been described.

## INTRODUCTION

Congenital orofacial teratoma, also known as epignathus, is an uncommon lesion estimated to affect between 1 in 35,000 and 1 in 200,000 live births.^1^^,^^2^ The tumor has the histological features of a mature, benign teratoma or immature, malignant teratoma, and is attached to an intraoral surface, most often palatal or pharyngeal.^3^ Occasional cases have been described with associated intracerebral teratoma, but this association is exceptional.^3^^-^^5^ Even more rare and exceptional is the circumstance of a teratoma with oropharynx destruction; in fact, only four cases have been described in the literature.

We describe the case of a 22- to 23-week gestational age (GA) fetus with pharyngeal mass only minimally protruding from the oral cavity that completely destroyed the oral cavity. The histological examination revealed an immature teratoma (G3); only one other G3 case has been described.

## CLINICAL REPORT

Here we report the prenatal case of a 22- to 23-week GA fetus of a 30-year-old Italian, primigravid woman and her nonconsanguineous, healthy 31-year-old husband. There was no reported exposure to teratogenic agents. Serologic tests for cytomegalovirus, toxoplasmosis, and rubella gave negative results. The family and early gestational history were unremarkable.

A morphological ultrasound scan, performed at GA 21 weeks and 3 days, showed a fetal malformation. A solid formation of 49 × 36 × 51 mm, moderately vascularized to the color Doppler extended from the lower part of the face to the upper part of the chest. The lower lip and mandibular bones, plus the esophagus and trachea were not recognizable. Posteriorly, the growth came into contact with the cervical spine. This formation presented the characteristics of a suspected malignant nature. The fetal and neonatal prognosis was very poor.

The pediatric consultant surgeon spoke of an injury that could not be surgically managed and was incompatible with life; thus, termination of the pregnancy was planned beyond 90 days GA according to Italian legislation. The molecular swab for Covid-19 was performed and was negative.

Fetus was well developed for its GA and was female (internal and external genital concordance).

**Figure 1:**
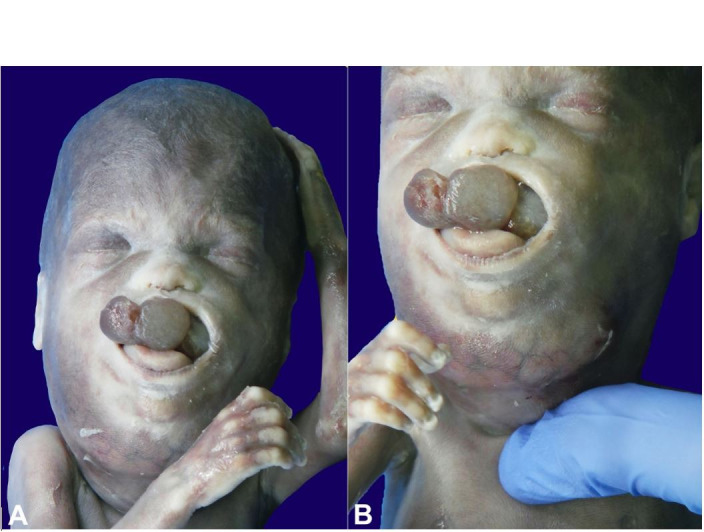
External examination. **A –** The lesion is minimally protruding from the mouth **B –** note the marked involvement of the neck.

Nose, nasal choanae, oral cavity, tongue, palate, and chin were affected by multilobate neoformation. The lesion extended throughout the oral cavity, involving the tongue that appeared incorporated in the mass; the tumor protruded to a small extent from the open mouth along with the tip of the tongue. The neck appeared very swollen due to the lesion itself, which extended its full length up to the jugular dimple; a subcutaneous venous reticulum was very evident (Figure 1).

**Figure 2:**
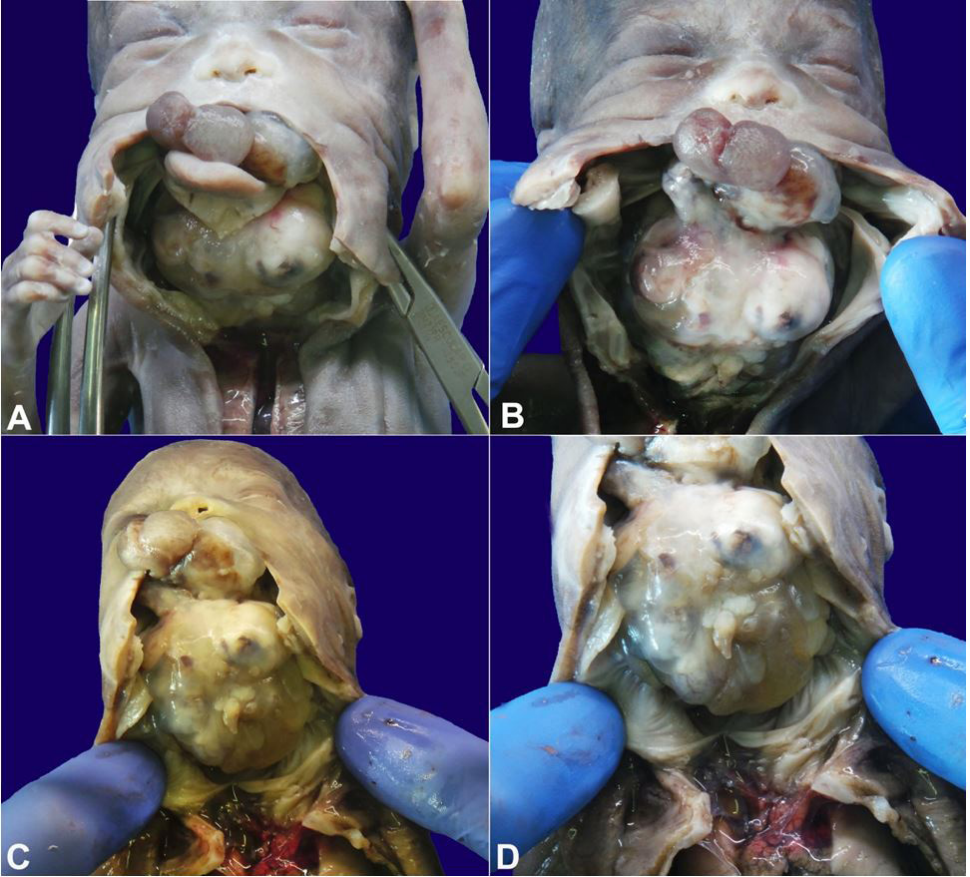
Complete view of the neoplasm. **A –** After a median cut (from the lower lip, to the jugular), the neoformation is seen extending throughout the oral cavity, and engulfing the tongue; **B –** View of the neoformation after removal of the tongue remnants; **C and D –** The lesion infiltrated the entire floor of the mouth, incorporating the tongue, with compression and antero-posterior displacement of the pharynx, larynx, and trachea. In particular, the root and body of the tongue were not recognizable because they were incorporated and destroyed by the neoplasm; only the apex of the tongue was identified

To remove the neoplasm, a median cutaneous incision was made until the neoformation was fully exposed. The tongue, hypopharynx, submandibular glands, larynx, esophagus, great vessels, lymph nodes, and thyroid gland were altered in position and shape due to the presence of a large neoplasm (Figure 2).

The neoformation was multilobate (6.5 × 4.5 × 4 cm; 38 g) mainly solid, fleshy, and whitish in color with greyish areas; it did not present cystic, hemorrhagic, or necrosis areas.

It was immediately evident that the lesion infiltrated the entire floor of the mouth, incorporating the tongue, with compression and antero-posterior displacement of the pharynx, larynx, and trachea. In particular, the root and body of the tongue were not recognizable because they were incorporated and destroyed by the neoplasm; only the apex of the tongue was identifiable. The floor of the mouth was completely destroyed, and the trachea and larynx were not discernible. The tumor only marginally reached the superior mediastinum.

**Figure 3:**
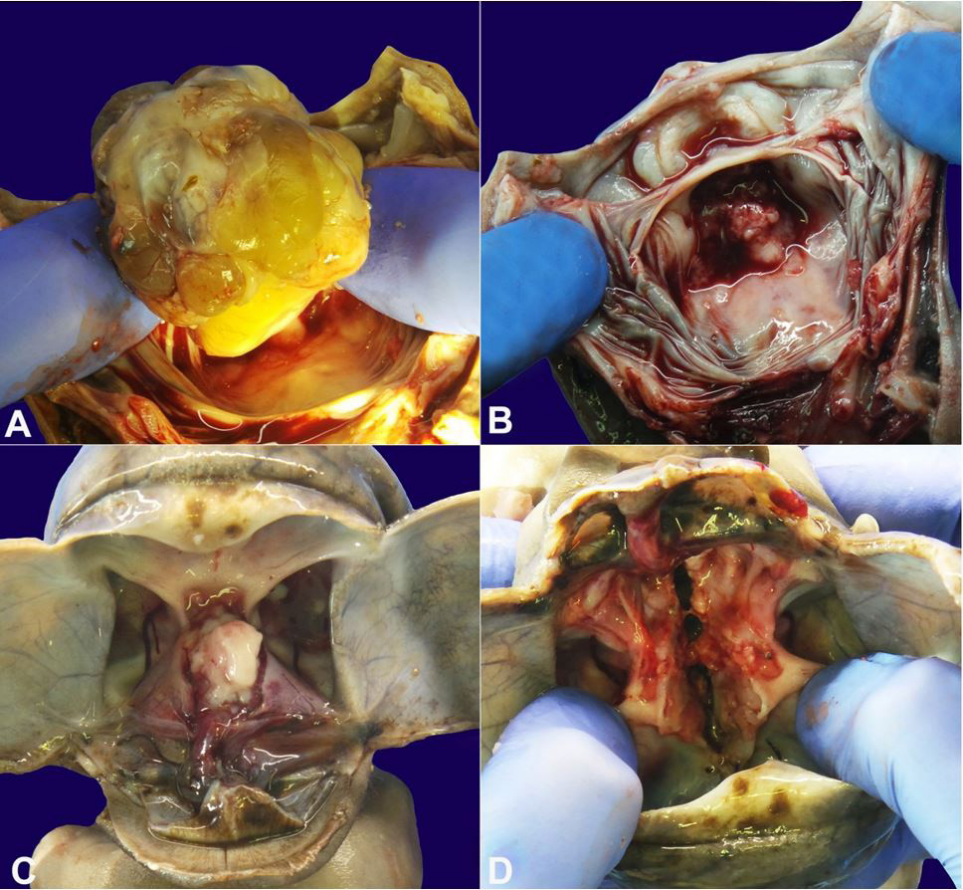
Intracranial component. **A –** The lesion was removed by cutting its peduncle anchored to the palate; **B –** The area of insertion of the lesion on the palate is exposed; **C –** After removal of the brain, the small intracranial part of the tumor could be identified; **D –** After a longitudinal cut of the sella turcica a communication can be seen between the epignathus and the intracranial component.

The neoformation, starting from the roof of the pharynx, extended throughout the pharynx (rhino and oropharynx) destroying the floor of the mouth, most of the tongue, larynx, and trachea. It protruded to a small extent from the mouth and infiltrated the sphenoid (straddling the pituitary dimple and the sella turcica) in a minimal portion (Figure 3).

**Figure 4:**
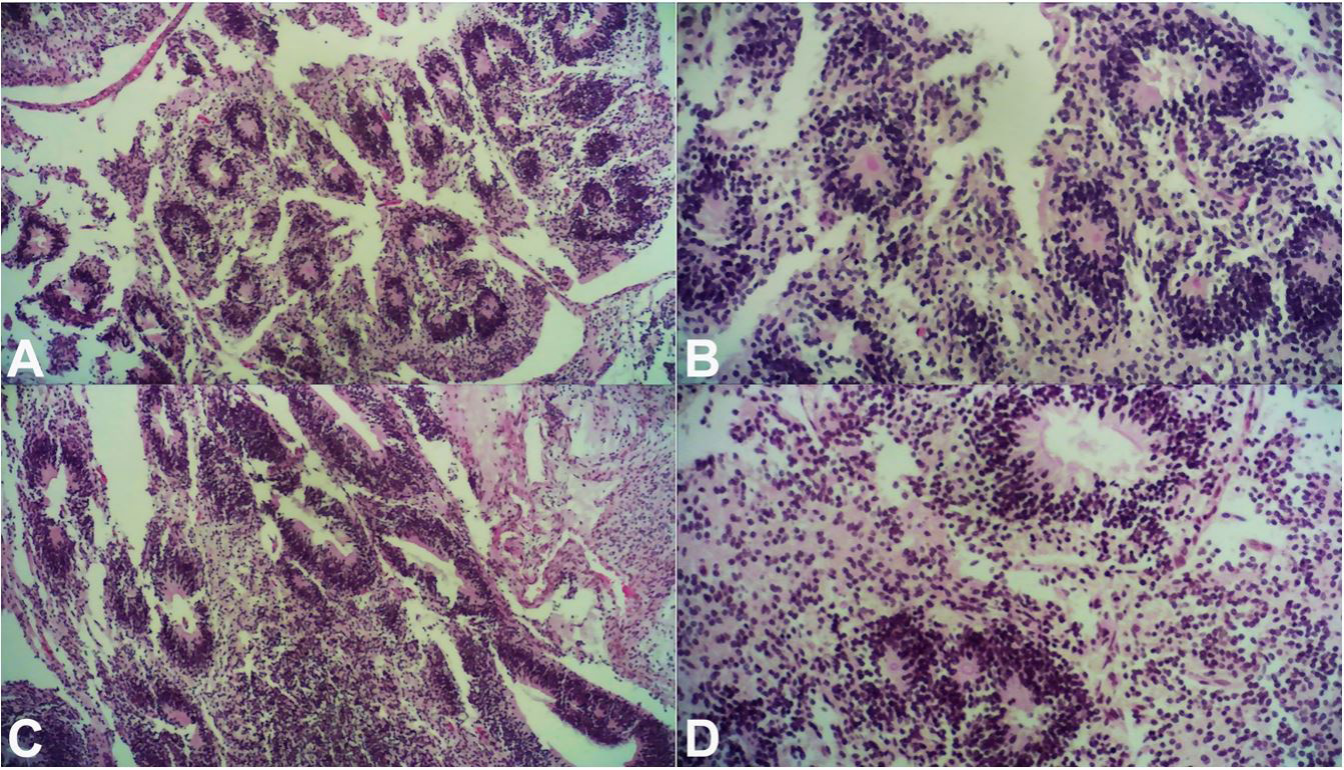
Microscopic images. The majority of the tumor is an immature teratoma composed of immature neuroepithelial tissue (A - H&E, 40X, B and C - H&E,100X, D - H&E, 200X).

Histological examination showed neoformation with growth at times infiltrative and destructive with only expansive growth. It was composed of immature tissues, largely tubules and neuroectodermal rosettes, interspersed with tissues of endodermal and ectodermal origin in various differentiation stages (Figure 4). The neuroectodermal tubules and rosettes were composed of mitotically active hyperchromatic cells.

Overall, the tumor had an abundant immature neuroepithelial tissue component occupying more than three low-magnification fields (40×) in each examined slide of the lesion. The neoplasm was therefore classified as a malignant neoplasm of the immature grade 3 or high-grade teratoma type. It was a particularly aggressive and rare lesion; in fact, approximately 12 surgically inoperable cases have been described in the medical literature.

## DISCUSSION

The word “teratoma” was first coined and defined by the famous scientist Virchow in the first edition of his book on tumors published in 1863. Teratomas range from benign to malignant, and solid to cystic. Teratoma arises from totipotent cells—the cells that give rise to ectoderm, endoderm, and mesoderm. These tumors typically are midline or paraxial with the most common location being sacrococcygeal (57%). Cystic teratomas occasionally occur in sequestered midline embryonic cell rests^6^^,^^7^ and can be mediastinal (7%), retroperitoneal (4%), cervical (3%), and intracranial (3%). Tumors arising from hard and soft palate and Rathke’s pouch are known as epignathus teratomas.

Clinically, a hard palate teratoma appears as a bulky, single mass or multiple little lesions with a pedicle or sessile. Airway obstruction is the main complication and is related to the size and site of the lesion occurring in 80% to 100% of cases.^8^ Differential diagnoses of neonatal oral lesion embrace embryonic congenital rhabdomyosarcoma, retinoblastoma, nasal glioma, heterotopic thyroid, cystic lymphangioma of the oro- or nasopharyngeal regions, and sphenoid meningoencephalocele.^9^

Most epignathus teratomas were identified during the second and third trimester by two-dimensional or three-dimensional ultrasound.^10^

In selected cases it is possible to remove the lesion and save the life of the newborn. Early radical removal is the treatment of large head and neck teratoma without an intracranial component.

There were reports of successful delivery of live fetuses by ex utero intrapartum treatment of the fetus with isolated epignathus.^11^

By studying all the English medical literature, we found 18 cases of epignathus with immature teratoma. This tumor always originates from the hard palate. In six cases the lesion also had an intracranial component^4^^,^^12^^-^^15^ (Table 1 and Table 2).

**Table 1:** Inoperable cases of epignathus.

**author**	**Mother**	**Size (mm)**	**wgt (g)**	**GA (w)**	**IC**	**Grade**	**Outcome**	**OD**
Our case	30 yo, 1G0P	46 × 36 × 51	38	22/3	**Yes**	3	Induced abortion	Yes
Kirishima et al. ^12^	32 yo, 3G2P	120 × 60 × 60	270	27	Yes	NR	Stillborn	No
Huang and Pan^16^	28 yo, 1G0P	55 × 41 × 28	NR	18	No	NR	Induced abortion	No
Wang et al.^13^	31 yo, 1G0P	67 × 65 × 50	NR	17/6	Yes	NR	Induced abortion	No
Faghfouri et al. ^17^	33 yo, 5G4P	153 × 108	NR	24/5	No	NR	Induced abortion	Yes
Nagy et al.^18^	22 yo, 2G0P	25 × 22	NR	21	No	NR	Induced abortion	Yes
Kumar et al.^19^	25yo, NR	95 × 75 × 60	NR	28–29	No	1	Succumbed to death immediately post-partum	No
Shertukde and Thelmo^20^	26 yo, 3G2P	150×90×70	645	26	No	NR	Asphyxia 45 min post-partum	Yes
Too et al.^14^	32 yo, 1G0P	80×100	NR	34	Yes	NR	5 weeks of life	Yes
Witters et al.^21^	31 yo, 7G0P	50	35	20	No	NR	Induced abortion	No
Alagappanet al.^22^	NR	140 × 160	810	26	Yes	NR	Perinatal death	No
Smith et al.^15^	30 yo, 3G1P	35	NR	29	Yes	NR	Induced abortion	No
	28 yo, 1G0P	20	NR	18	Yes	NR		No
Ducket and Claireaux^4^	26 yo, 2G1P	NR	50	31 w	Yes	NR	55 min post-partum	No

g= gram; GA= gestational age; IC= intracranial component; NR= not reported; OD= oropharynx destruction; w= week; wgt= weight;

**Table 2:** Cases of epignathus undergoing surgery.

**author**	**Mother**	**Size (mm)**	**wgt (g)**	**GA**	**IC**	**Grade**	**Outcome**	**OD**
Izadi et al.^23^	29 yo, 3G2P	160 × 200 × 60	371	29 w	No	NR	Successful excision	No
Prevedello et al.^24^	NR, 3G2P	68 × 65 × 62	NR	33 w	No	NR	Successful excision	No
Sumiyoshi et al.^25^	23 yo, NR	100 longest axis	246	28/5 w	No	NR	Successful excision	No
Rayudu et al.^26^	NR	90 × 100	NR	NR, full term pregnancy, 2 days old neonate	No	NR	Successful excision	No
Chung et al.^27^	29 yo, 1G0P	150 × 90 × 60	392	27/5 w	No	3	Successful excision	No
Ince et al.^11^	24 yo, 1G1P	130 × 110 × 90	545	33/1 w	No	NR + Nephroblastoma component	NR	No

g= gram; GA= gestational age; IC= intracranial component; OD= oropharynx destruction; NR= not reported; w= week; wgt= weight.

Much more rarely, only in four cases,^17^^,^^18^^,^^20^^,^^27^ as in our case, the lesion extended from the nasopharynx to the oropharynx and even up to the hypopharynx. Only in our case was the complete destruction of the tongue and the floor of the mouth. The degree of malignancy was reported only in two of the many cases described;^19^^,^^27^ only one case was grade 3 (as in the case we describe).

This kind of lesion frequently has a wide component that bulges from the oral cavity. In our case, the extra-oral component was small; however, the oropharyngeal and hypopharyngeal components were very extensive with expansive and destructive growth. The particularities of the case we described are the high degree of malignancy, the destructive oropharyngeal and hypopharyngeal growth, and the scarce extra-oral component.

## CONCLUSION

Congenital hard palate teratomas or epignathus teratomas are rare tumors that can be diagnosed during gynecological visits by a simple investigation modality such as ultrasonography. The correct plan can be made to terminate the pregnancy in cases of large and fatal lesions with a high rate of morbidity and mortality.

The case we describe is very rare. When a fast-growing fetal epignathus is detected early, pregnancy termination should be measured. In selected cases it is possible to plan the resection of the lesion; unfortunately, in our case, this was not possible.^11^^,^^23^^-^^26^
